# Still Another Death from Chloroform—Shall Its Use Continue?

**Published:** 1861-02

**Authors:** 


Still Another Death from Chloroform—Shall its Use Continue?—A death from chloroform, at the Northampton Infirmary is recorded in the London *Medical Times and Gazette*. The patient was about to submit to an operation for the removal of a small tumor from the back. Chloroform was administered cautiously. The testimony before the coroner's jury says that the effects of the anæsthetic were soon visible upon the deceased, who became insensible without anything unusual being observed, although he was closely watched. On removing him into a proper position for performing the operation, it was observed that his countenance was very much changed. The suspicions of the operators were at once roused, and immediate steps were adopted for bringing the man to his senses again, instead of commencing the surgical operation. Restoratives were resorted to, but to no purpose. Artificial respiration was then attempted, but this, too, was unavailing, and after an hour's futile endeavors at respiration, the deceased was reluctantly given up as lost.

We record this case, not from any peculiar interest that it possesses, but that it may be added to the long, dark list which now stands against chloroform. All statistics of fatalities from chloroform which we have seen, are inaccurate and far below the truth in their estimates, and we desire that every fatal case attributable to it may hereafter be presented. It is hoped that such cases, instead of being concealed, as they often have been, as if the unfortunate administrator felt guilty of homicide, will henceforth be published. We believe that, had all the deaths from chloroform been properly noted, a list could now be presented which would astonish most of the advocates of its use, and do much in future to prevent loss of life by causing the discontinuance of its administration.

The practice of administering chloroform is rapidly decreasing in this country, and we sometime ago predicted its discontinuance for general anæsthetic purposes, and the substitution of ether. If its use is not lessening in Europe, there is a growing want of confidence in its safety. The very caution with which European surgeons give it, shows that they use it with a consciousness of its danger. In the testimony of this case it was stated that the operator had the precaution to examine the deceased, "to ascertain if he was able to bear the effects of the chloroform." In the use of ether, which is now admitted to be almost absolutely safe, no one ever thinks of using such precautions, and it is now deemed admissable in any condition in which its anæsthetic effects are desirable.

We relinquished the convenient use of chloroform with reluctance. When deaths under its administration became frequent, we still hoped that greater caution in its use, increased study of its physiological effects, further knowledge of the conditions which contra indicate it, and a discovery of the constitutional idiosyncracies which make the administration fatal, would enable us to continue its use with safety. But the fatalities are now so numerous, as unexpected, and as inexplicable as ever. Patients in vigorous health, and under the most cautious hands, continue to die when but little of the vapor has been inhaled, and sometimes at almost the first inspiration.

In the present state of our knowledge of the mysterious fatal influences of chloroform and its acknowledged uncontrollable mortality, we consider its ordinary use unjustifiable while an efficient and safe alternative for use is at hand. If the profession do not discontinue its use, patients will soon refuse it. There is already, owing to its dangers, an increasing predjudice among the masses in regard to anæsthesia, in whatever manner produced, and if the use of chloroform, with its fatal accompaniments, continues, the popular verdict will condemn anæsthetics entirely, preferring to suffer pain rather than incur such a hazard of life.—*Medical and Surgical Reporter.*

